# Amiodarone-Related Pure Red Cell Aplastic Anemia and Hypothyroidism in a Child With Total Anomalous Pulmonary Venous Connection

**DOI:** 10.3389/fped.2019.00361

**Published:** 2019-09-03

**Authors:** Hongjun Ba, Lingling Xu, Huimin Peng, Xuandi Li, Yuese Lin, Huishen Wang, Youzhen Qin

**Affiliations:** ^1^Department of Pediatric Cardiology, Heart Center, The First Affiliated Hospital, Sun Yat-sen University, Guangzhou, China; ^2^Department of Pediatrics, The First Affiliated Hospital, Sun Yat-sen University, Guangzhou, China

**Keywords:** pure red cell aplastic anemia, amiodarone, hypothyroidism, congenital heart disease, prednisone

## Abstract

**Introduction:** Amiodarone is an effective anti-arrhythmic drug, but there are many clinical side effects that limit its application. There are no case reports of amiodarone-related pure red cell aplastic anemia (PRCA).

**Case Presentation:** Here, we present a case of amiodarone-related PRCA and hypothyroidism in a 7-month-old boy. The patient had a total anomalous pulmonary venous connection (the cardiac type) and had undergone cardiac surgery at the age of 2 months. Eleven days after the operation, atrial tachycardia was observed. Amiodarone was administered orally (15 mg/kg.d), following which the arrhythmia was under control. Subsequently, the patient was prescribed amiodarone (5 mg/kg.d) and discharged. Regular medical consultations were not conducted as required. At 7 months of age (5 months after the operation), the patient returned to the hospital for re-examination. The electrocardiogram showed intermittent sinus bradycardia, occasional junctional escape beats, hemoglobin 7.9 g/DL, and thyroid function—TSH 9.660 uIU/mL.

**Results:** Amiodarone was discontinued. Thyroxine was administered orally. Subsequently, the heart rate improved and TSH returned to normal levels. Nutritional therapy was recommended based on a diagnosis of nutrition-related anemia. A re-visit at 9 months of age showed that the weight was 6 kg, but the routine blood test indicated that hemoglobin was 5.9 g/DL with positive cell anemia and low reticulocyte count. Bone marrow cytology examination suggested PRCA. The hemoglobin level was gradually restored after treatment with prednisone.

**Conclusion:** The use of amiodarone in small infants and young children and its side effects should be carefully monitored. The potential mechanism of amiodarone-related PRCA needs further study.

## Introduction

Amiodarone is a class III, broad-spectrum, anti-arrhythmic drug that is highly effective in treating both atrial and ventricular arrhythmias (1). However, it is associated with a wide variety of side effects that limit its clinical application. Adverse effects include thyroid dysfunction, pulmonary fibrosis, optic neuritis, ataxia, and hepatitis (2–4). Hematologic side effects include bone marrow granulomas, pancytopenia, hemolytic anemia, neutropenia, and thrombocytopenia (5–8). Amiodarone-related aplastic anemia is very rare, and to the best of our knowledge, only one such case has been reported in an adult (9). We describe a pediatric patient who developed pure red cell aplastic anemia (PRCA) and hypothyroidism during amiodarone therapy.

## Case Report

A 7-month-old, Chinese, male patient was referred to our center for post-operative evaluation of total anomalous pulmonary venous connection (the cardiac type, with anomalous connections to the coronary sinus), which was diagnosed and operated at the age of 2 months. The patient was born after a full-term gestation, from non-consanguineous parents and the weight at birth was 3.7 kg.

The pre-operation body weight was 4.1 kg. Eleven days after the operation, atrial tachycardia was observed. Maximum heart rate was about 200 beats per minute. Amiodarone was administered orally (15 mg/kg.d), and subsequently, the arrhythmia was under control. Amiodarone was reduced to 10 mg/kg.d after 4 days and to 5 mg/kg.d after 1 week. The patient was discharged with a prescription for amiodarone (5 mg/kg.d). Regular medical consultations were not conducted as required.

Physical examination of the child at 7 months of age showed that his weight was 4 kg (3 standard deviations below the mean) and height, was 62 cm (3 standard deviations below the mean). At rest, his heart rate was slow−80 beats per minute. Blood exams showed that hemoglobin was 7.9 g/DL with positive cell anemia, and thyroid function: TSH 9.660 uIU/mL(normal reference range: 0.5–5 uIU/mL). Serum ferritin, serum iron, folic acid, and vitamin B12 were all detected at normal levels. The serum bilirubin was not high, and the urobilinogen and hemolytic tests were all negative. The electrocardiogram showed intermittent sinus bradycardia with occasional junctional escape beats. These symptoms were diagnosed as the side effects of excess amiodarone. As a result, it was discontinued. Thyroxine was administered orally. Subsequently, the heart rate improved, and TSH level returned to normal.

**Figure 1:**
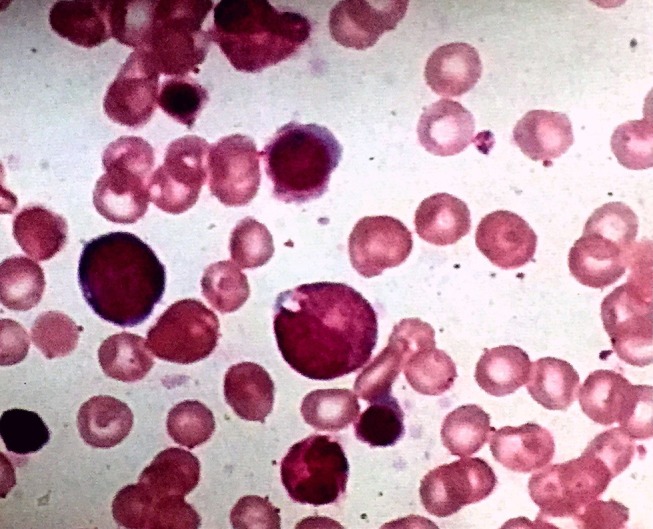
Bone marrow smear (×100 magnification) showing normal trilineage hematopoiesis with the presence of erythroid precursors.

**Figure 2:**
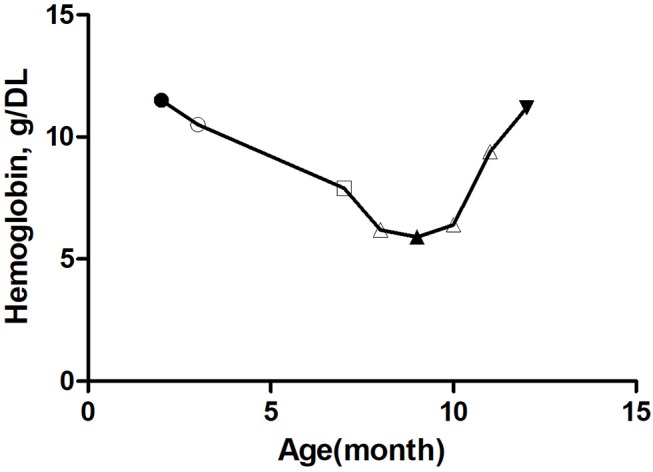
Trends of hemoglobin with age in this patient. 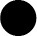 Preoperative hemoglobin, 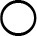 Hemoglobin before taking amiodarone, 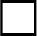 Hemoglobin after 4 months of amiodarone administration, 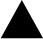 Hemoglobin before treatment with prednisone, 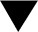 Hemoglobin after 3 months of prednisone administration. △ The first one: Hemoglobin 1 month before amiodarone administration, The second one: Hemoglobin 1 month after amiodarone administration, The third one: Hemoglobin 2 months after amiodarone administration.

Re-examination at 8 months of age showed that the weight had increased by 1.3 kg; TSH was normal but the child was anemic and hemoglobin was 7.0 g/DL. Since the patient was underweight, nutritional therapy was recommended. A re-visit at 9 months of age showed that the weight was 6 kg, but a routine blood test indicated that hemoglobin was 5.9 g/DL with positive cell anemia and low reticulocyte count. Bone marrow cytology examination suggested PRCA (Figure 1). The parents denied that the child had been exposed to drugs such as chloramphenicol and ampicillin that could cause aplastic anemia. All tests were negative, including cytomegalovirus, Epstein-Barr virus, and parvovirus B19. There was no family history of anemia. Prednisone was administered orally (2 mg/kg.d). Regular follow-up in pediatric clinics, every 2–4 weeks, was recommended. Two weeks after treatment with prednisone, the hemoglobin increased to 8.2 g/DL. After 4 weeks, the hemoglobin further increased to 11.2 g/DL (Figure 2). Two months after prednisone treatment, prednisone dosage was reduced to 0.5 mg/kg.d.

At present, the child is 1 year old, 7 kg in weight, and 67 cm in height. Five months after treatment with prednisone), the hemoglobin was 11.3 g/DL (Figure 2). Thyroid function was normal. The current dose of prednisone is 2.5 mg per day.

## Discussion

PRCA syndrome is defined by severe reticular cell reduction, a significant reduction in or lack of bone marrow erythrocyte precursors, and cell-less normal anemia (10). PRCA includes congenital and acquired pure erythrocytes. Acquired PRCA may be related to lymphoblastic diseases, especially chronic lymphoblastic leukemia; autoimmune disorders; infections, especially the B19 viral infection; pregnancy; hematologic malignancies; non-hematologic neoplasms; and drugs and toxins (10). It is reported that there are many drugs that cause PRCA such as ampicillin, carbamazepine, etc (11, 12). However, there are no reports of amiodarone-related PRCA.

Amiodarone-related blood system damage is reported more often but reports of anemia are rare. Our patient had no anemia before and immediately after the operation. After the use of amiodarone, positive cell anemia occurred, white blood cells and platelets were normal, reticular red blood cells were reduced, and bone marrow examination suggested PRCA. Although amiodarone was discontinued, the anemia continued to worsen in our patient for 2 months, and this may be due to the extremely large volume of distribution and long half-life of amiodarone. Anemia improved after the patient was treated with prednisone. This is consistent with the assumption that acquired PRCA is associated with immune disorders (10). Major factors that lead to amiodarone-related side effects include long-term high-dose usage and low body weight (13). This patient had complex, congenital heart disease, low weight, and long-term usage of the drug due to irregular follow-up. Therefore, the use of amiodarone in low-weight children, especially for long periods, should be carefully monitored.

In addition to PRCA, our patient had hypothyroidism. There have been several reports of side effects of amiodarone-related thyroid function impairment (14–16). Thus, it is consistent with the amiodarone-related side effects. Moreover, the child showed bradycardia in the electrocardiogram. After discontinuation of amiodarone therapy, the electrocardiogram became normal. There was a significant increase in weight after thyroid hormone replacement therapy. At present, the mechanism of amiodarone-related PRCA is unclear and needs to be further studied.

In conclusion, doctors should be careful regarding the use of amiodarone in young and low-weight children and avoid long-term use. Side effects should be closely monitored during use.

## Data Availability

All datasets generated and analyzed in this study are included in the manuscript and the supplementary files.

## Ethics Statement

Written informed consent was obtained from patients' parents for publication of this case report and any potentially identifying information. The work was exempt from the ethics committee review/approval.

## Author Contributions

All authors listed have made a substantial, direct and intellectual contribution to the work, and approved it for publication.

### Conflict of Interest Statement

The authors declare that the research was conducted in the absence of any commercial or financial relationships that could be construed as a potential conflict of interest.
